# The role of electrocardiogram in sex verification in a young adult with primary amenorrhea: a case report

**DOI:** 10.1186/s13256-018-1793-x

**Published:** 2018-09-17

**Authors:** Muritala A. Asafa, Rahman A. Bolarinwa, Omotayo A. Eluwole, Bolanle O. Ibitoye, Adesoji M. Adegoke, Oluwadare Ogunlade

**Affiliations:** 1 Department of Physiological Sciences, Faculty of Basic Medical Sciences, College of Health Sciences, Obafemi Awolowo University, Ile-Ife, Nigeria; 2 Department of Haematology and Immunology, Obafemi Awolowo University and Teaching Hospital, Ile-Ife, Nigeria; 3 Department of Medical Pharmacology and Therapeutics, Obafemi Awolowo University, Ile-Ife, Nigeria; 4 Department of Radiology, Obafemi Awolowo University, Ile-Ife, Nigeria; 5 Science Central Laboratory, Obafemi Awolowo University, Ile-Ife, Nigeria

**Keywords:** Electrocardiogram, Sex verification, Amenorrhea, Karyotyping, Young adult

## Abstract

**Background:**

The use of electrocardiogram for sex verification in adults is an emerging concept in medicine. It is feasible through the utilization of Ogunlade Sex Determination Electrocardiographic Score. The aim of this study was to use an electrocardiogram to verify the sex of a woman with primary amenorrhea.

**Case presentation:**

We report a case of a 36-year-old woman of Yoruba ethnicity who presented with primary amenorrhea. A physical examination revealed a woman with a feminine appearance characterized by long plaited hair and well-developed breasts. As part of the investigations to unravel the sex status, she had a resting standard 12-lead electrocardiogram which revealed a masculine electrocardiogram pattern with Ogunlade Sex Determination Electrocardiographic Score of 9 (T-wave pattern in lead V_1_, 3; ST segment in lead V_2_ or V_3_, 3; QRS rotation, 2; heart rate of 79, 1). An abdominopelvic ultrasonography done by a radiologist showed absence of uterus, fallopian tubes, and ovaries. When our patient was considered for transvaginal scan, she declined but embraced translabial ultrasound as she claimed to be a virgin. Translabial ultrasonography revealed the presence of undescended hypoplastic testes with associated testicular microlithiasis at the external inguinal rings bilaterally. Karyotyping using a blood sample revealed 46,XY and a sex-determining region Y report showed that the blood sample was positive for the *SRY* gene confirming the status as male. This synchronized with the initial electrocardiogram evaluation. The testes were later removed.

**Conclusion:**

This report concluded that an electrocardiogram as a cheap, readily available and non-invasive test has a role in sex verification in young adults with primary amenorrhea.

## Background

Sex verification in the young especially among people with suspected sex ambiguity or reproductive challenge or suspected masquerades in sport is tasking and sensitive. Various approaches including physical appearance and genetic sex testing have been utilized. The standard tests are chromosome-based verification [1]. However, chromosomal-based analyses are expensive and, in addition to some difficult ethical considerations, they are not readily available [2]. There is therefore a need for evaluation of other methods for sex verification. A biological signal that has been considered to have potential for sex verification in the young is the electrocardiogram (ECG) [3]. Sex differences in ECG have been coded into a point scoring system called Ogunlade Sex Determination Electrocardiographic Score (OSDES). The scoring system utilizes four ECG parameters (Table 1). A total OSDES of < 7 identifies female while a score ≥ 7 identifies male. The scoring system was evaluated and found to have a reasonable degree of sensitivity and specificity [4]. This special use of ECG is a new development and advancement over the traditional interpretation of ECG for identification of cardiac and extracardiac disorders [5]. This present study evaluated the role of ECG in determining the sex status of a person presenting with absence of menses at age of 36 years.

**Table 1:** Ogunlade Sex Determination Electrocardiographic Score

Parameters		Criteria	Score
1	T wave in V1	Inversion	1
		Flattened	2
		Upright	3
2	ST segment in V2 or V3	Isoelectric: at 0–1 mm of PR segment	1
		Depression: ≥ 2 mm below PR segment	2
		Elevation: ≥ 2 mm above PR segment	3
3	Rotation	Anticlockwise rotation	1
		Normal transition	2
		Clockwise rotation	3
4	Heart rate (beats per minute)	> 70	1
		60–70	2
		< 60	3
Total			4–12

Ogunlade Sex Determination Electrocardiographic Score < 7 = female, Ogunlade Sex Determination Electrocardiographic Score ≥7 = male

## Case presentation

**Fig. 1:**
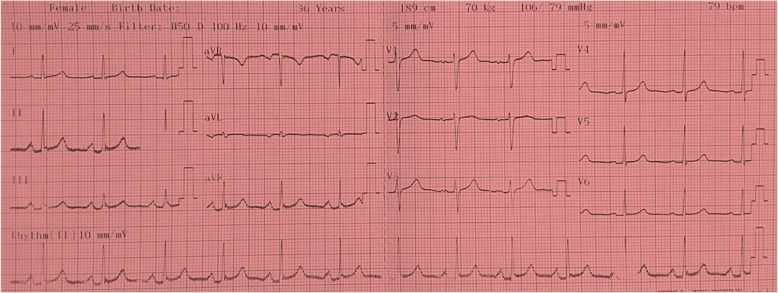
Electrocardiogram of a 36-year-old woman. T-wave pattern in lead V_1_ = 3, ST segment in lead V_2_ or V_3_ = 3, QRS rotation = 2, heart rate of 79 = 1. Ogunlade Sex Determination Electrocardiographic Score = 9

**Fig. 2:**
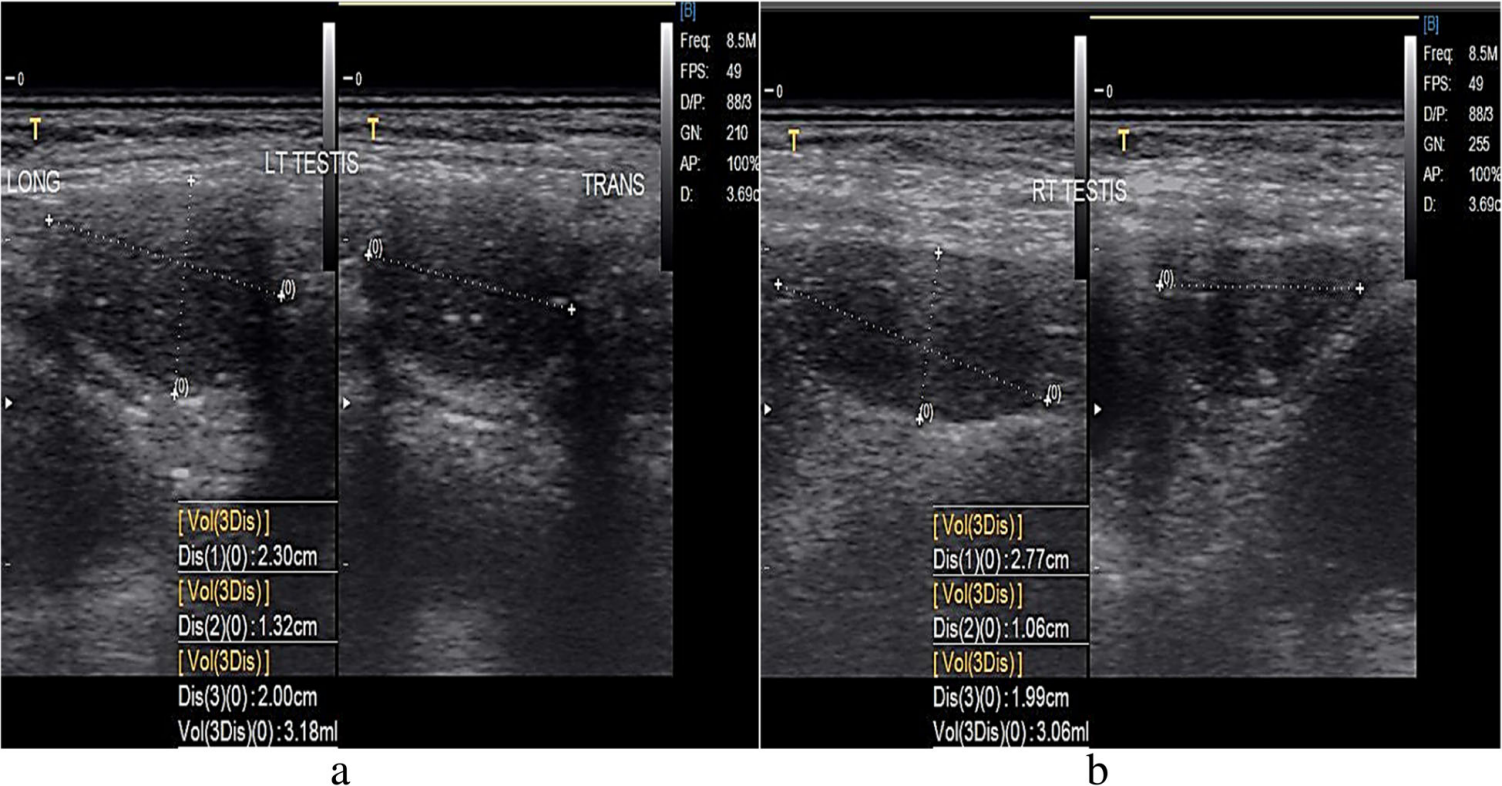
Translabial ultrasonographic pictures of right and left testes. Left and right testes located at the external inguinal ring. The right testis (**b**) is slightly smaller in volume than the left testis (**a**). *LT* left, *RT* right

**Fig. 3:**
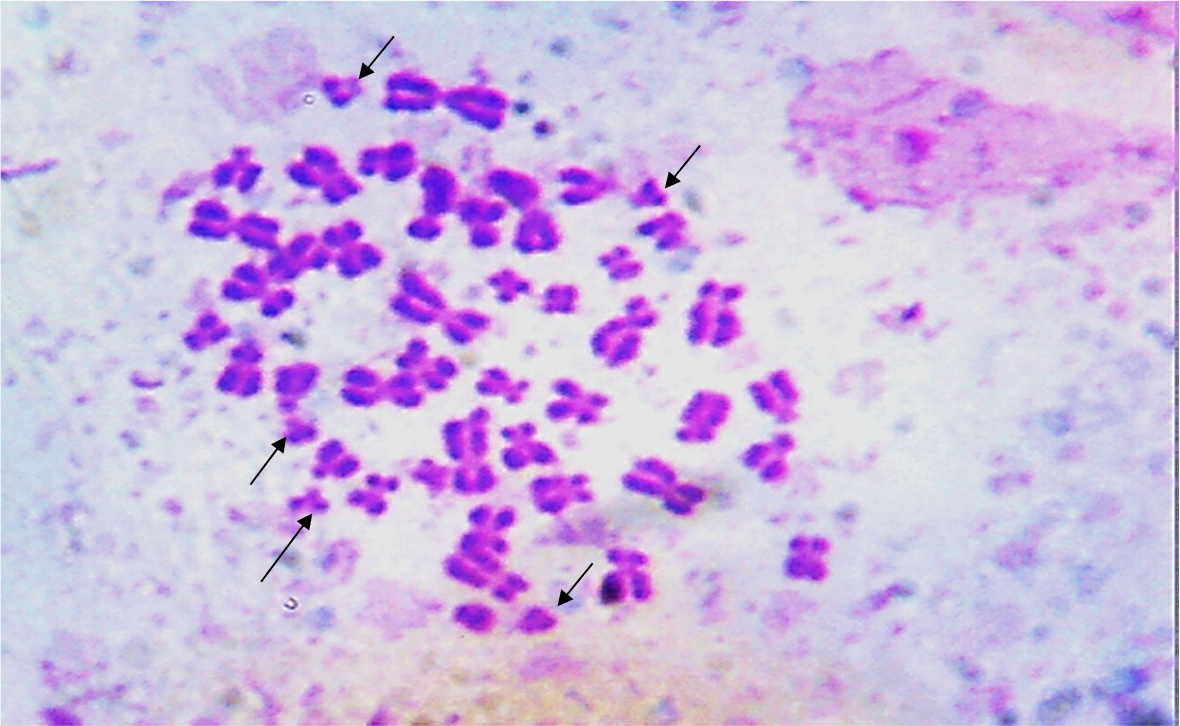
Chromosomal analysis of the patient. The chromosomal sex of the patient is male (46, XY) as the *arrows* indicate. No gross chromosomal abnormalities involving structural or number changes were observed. The *arrows* indicate the 5Gs

**Fig. 4:**
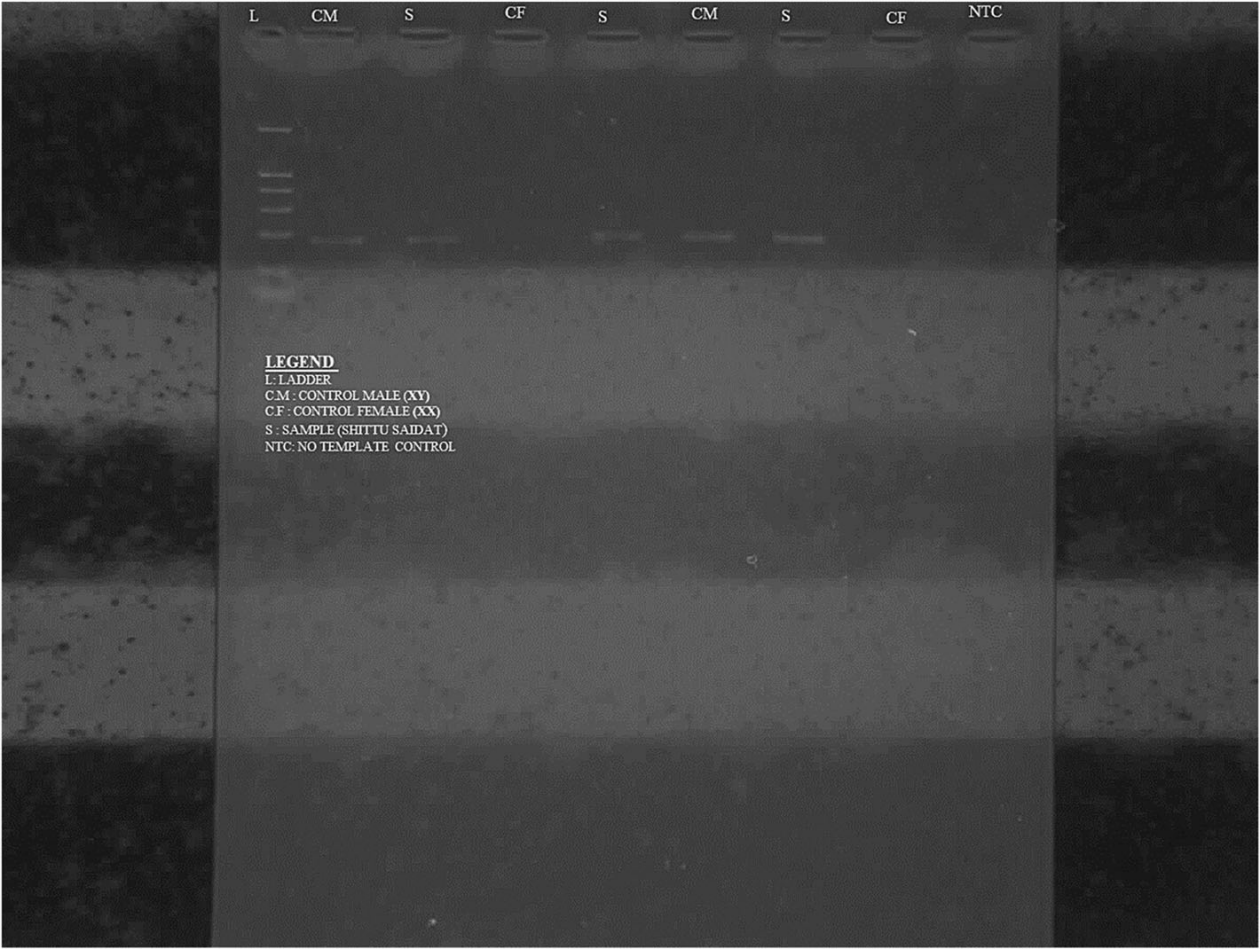
Sex-determining region Y. Y-chromosome material was found in the sample of the patient when compared with that of an already known male

We report a case of a 36-year-old woman of Yoruba ethnicity who presented with absence of menses. A physical examination revealed a woman with feminine appearance characterized by long plaited hair and well-developed breasts. As part of the investigations to unravel the sex status, she had a resting standard 12-lead ECG following standard protocol. The ECG was in sinus rhythm and it revealed a masculine electrocardiographic pattern with OSDES score of 9 (T-wave pattern in lead V_1_, 3; ST segment in lead V_2_ or V_3_, 3; QRS rotation, 2; heart rate of 79, 1) as shown in Fig. 1. An abdominopelvic ultrasonography done by a radiologist showed absence of uterus, fallopian tubes, and ovaries. When our patient was considered for transvaginal scan, she declined but embraced translabial ultrasound as she claimed to be a virgin. Translabial ultrasonography revealed the presence of undescended hypoplastic testes with associated testicular microlithiasis at the external inguinal rings bilaterally (Fig. 2a, b). The karyotyping done using blood revealed no gross chromosomal abnormalities involving structural or number changes. The chromosomal sex of male (46,XY) was reported (Fig. 3). The sex-determining region Y (*SRY*) was also done using QIAmp Blood Mini Kit (Qiagen) by extracting deoxyribonucleic acid (DNA) from a peripheral blood sample of our patient. Polymerase chain reaction was done with a pair of *SRY* forward (tacaggccatgcacagagag) and reverse (tcttgagtgtgtggctttcg) primers and Taq DNA polymerase with the use of appropriate positive and negative controls. Electrophoresis of the polymerase chain reaction product was done in 2% agarose gels and the bands visualized under ultraviolet (UV) light. The result showed that the blood sample was positive for the *SRY* gene which further confirmed the male status (Fig. 4). The genetic sex testing result confirmed initial electrocardiographically determined sex status of our patient. She was counseled and later had the testes removed to prevent malignant transformation.

## Discussion

Initially, sex verification took the form of physical examinations. It subsequently evolved into chromosome testing, and later testosterone testing. It is not always a simple case of checking for XX versus XY chromosomes, or sex hormone levels, to determine whether an athlete is unambiguously a woman or a man. Fetuses start out as undifferentiated and the Y chromosome turns on a variety of hormones that differentiate the baby as a male. Sometimes this does not occur, and people with two X chromosomes can develop hormonally or phenotypically as a male, and people with an X and a Y can develop hormonally or phenotypically as a female [6].

The verification of sex especially among athletes has been an issue for many decades prior to the introduction of chromosomal analysis. When sex verification was first introduced in international sporting competition during the early 1960s, female athletes underwent physical examinations where they stood naked before a committee of experts, this was known as “nude parade” [7]. The method of nude parade was later widely resented. Sex chromatin testing (buccal smear) was introduced at the Mexico City Olympic Games in 1968. It subsequently evolved into chromosomal analysis which is known to be the gold standard. The method is invasive, expensive, and not readily available in most developing countries [4]. ECG is a graphical record of the electrical activities of a heart obtained on the body surface. It is a basic non-invasive investigation with great application in medical practice for assessment of heart diseases especially heart blocks and cardiac arrhythmias [8]. It is one of the most commonly conducted cardiovascular diagnostic procedures in clinical practice [9]. In this index patient, the ECG-determined sex status was in total agreement with genetic sex testing. This implied that ECG, being a cheap readily available and non-invasive test, can be useful in sex verification in conditions of sexual ambiguity and primary amenorrhea.

## Conclusion

ECG as a cheap, readily available, and non-invasive test has a role in sex verification in young adults with amenorrhea and could be adopted in sports as a quick method to ascertain the sex of adults.

## Abbreviations

ECG: Electrocardiogram
OSDES: Ogunlade Sex Determination Electrocardiographic Score
SRY: Sex-determining region Y
UV: Ultraviolet
